# Pregnancy Complicated by 3-Hydroxy-3-Methylglutaryl-CoA Lyase Deficiency and Kaposiform Lymphangiomatosis

**DOI:** 10.1097/og9.0000000000000147

**Published:** 2026-01-29

**Authors:** Catherine Yang, Antonio Saad, Guoyang Luo

**Affiliations:** Department of Obstetrics and Gynecology, Inova Fairfax Hospital, Falls Church, Virginia.

## Abstract

**BACKGROUND:**

To describe pregnancy complicated by deficiency of 3-hydroxy-3-methylglutaryl–CoA (HMG-CoA) lyase, an inborn error of metabolism, and kaposiform lymphangiomatosis (KLA), a lymphatic anomaly with poor prognosis.

**CASE:**

A 24-year-old woman with known HMG-CoA lyase deficiency and KLA presented at 6 weeks of gestation with severe vomiting and dehydration. Her pregnancy was complicated by worsening lung disease, fetal growth restriction, anemia, thrombocytopenia, and gestational diabetes. She required parenteral nutrition and adjustments in immunosuppressive therapy. At 37 weeks, she underwent induction of labor and delivered a male infant weighing 2,790 g with reassuring Apgar scores.

**CONCLUSION:**

This case demonstrates that, with multidisciplinary management, patients with coexisting rare disorders can achieve successful pregnancy outcomes despite substantial maternal and fetal risks.

## Abstract 2

Successful pregnancy is possible in patients with 3-hydroxy-3-methylglutaryl–CoA lyase deficiency and kaposiform lymphangiomatosis when managed with multidisciplinary care, nutritional support, and individualization of standard care.

**Teaching Points**

1. Patients with multiple rare diseases such as 3-hydroxy-3-methylglutaryl–CoA (HMG-CoA) lyase deficiency and kaposiform lymphangiomatosis can achieve favorable pregnancy outcomes with appropriate management with a multidisciplinary team.
2. Patients with HMG-CoA lyase deficiency are particularly vulnerable to metabolic decompensation in early pregnancy, which can be avoided with timely care and aggressive nutritional support.
3. Patients with kaposiform lymphangiomatosis should be monitored closely for disease and symptom progression with immunosuppressant doses adjusted accordingly with fetal monitoring for in-utero exposure of immunosuppressants.

Deficiency of 3-hydroxy-3-methylglutaryl (HMG)-CoA lyase is a rare autosomal recessive disorder affecting ketone body metabolism as a result of *HMGCL* gene mutations.^1^ Affected individuals with homozygous genotypes cannot process leucine, preventing ketone production during fasting. Most are diagnosed in infancy with vomiting, hypoglycemia without ketosis, and decreased consciousness, which can be fatal.^2^ Metabolic crises may occur as a result of stress, illness, or prolonged fasting, leading to hyperammonemia, metabolic acidosis, and liver dysfunction.^2^ Management includes avoiding fasting, a low-fat/mildly protein-restricted diet, and carnitine supplementation.^3^ In the seven reported pregnancies, outcomes included four live births (one vaginal delivery, three cesarean deliveries), one spontaneous abortion at 10 weeks, one elective termination at 6 weeks, and one maternal–fetal death at 9 weeks.^4–6^

**Table 1.** Clinical Considerations in Pregnancy Complicated by Kaposiform Lymphangiomatosis and 3-Hydroxy-3-Methylglutaryl–CoA Lyase Deficiency

Domain	KLA Considerations	HMG-CoA Lyase Deficiency Considerations
Maternal risks	Progressive respiratory compromise attributable to thoracic disease and increased maternal oxygen demand High risk of severe hemorrhage if baseline consumptive coagulopathy (thrombocytopenia, hypofibrinogenemia) present	High risk of metabolic decompensation during fasting, illness, decreased appetite (eg, hyperemesis), labor, or postpartum Hypoketotic hypoglycemia, metabolic acidosis, hyperammonemia, and encephalopathy possible
Fetal risks	Fetal growth restriction and preterm birth resulting from maternal hypoxemia or instability Possible effects of in utero sirolimus exposure (low birth weight, neonatal cytopenia)	Risk of fetal growth restriction and fetal compromise secondary to maternal metabolic crises Autosomal recessive inheritance: 50% recurrence risk if father is a carrier, 100% unaffected carriers if father is not a carrier
Medical therapy	Sirolimus is the first-line agent, but safety in pregnancy is uncertain; requires individualized risk–benefit assessment	Strict avoidance of fasting High-carbohydrate, low-fat diet with carnitine supplementation IV glucose during illness, labor, and postpartum
Maternal–fetal surveillance	Regular respiratory assessments; coagulation studies to detect evolving consumptive coagulopathy Serial ultrasound (every 4 wk) for fetal growth	Maternal metabolic monitoring (glucose, acid–base status, ammonia, liver function tests) Serial ultrasound for fetal growth (every 4 wk)
Delivery planning	Tertiary care center with maternal critical care, transfusion services, and NICU Anticipate massive transfusion (red blood cells, platelets, cryoprecipitate/fibrinogen, antifibrinolytics) Delivery mode individualized	Delivery in tertiary center with metabolic expertise Continuous IV glucose (10%) during labor and 24–48 h postpartum Avoid prolonged fasting, including preoperative NPO periods
Anesthesia/procedural concerns	Neuraxial anesthesia contraindicated with coagulopathy or thrombocytopenia General anesthesia may worsen respiratory compromise; requires advance planning	Regional anesthesia preferred if coagulation is normal IV glucose required perioperatively; minimize fasting
Postpartum management	High risk of postpartum hemorrhage; close coagulation and hemodynamic monitoring Consider sirolimus resumption postpartum if withheld	High risk of metabolic crisis postpartum; maintain IV glucose until oral intake adequate Consider lactation suppression to avoid catabolic stress Neonatal evaluation for metabolic disease if at risk

KLA, kaposiform lymphangiomatosis; HMG-CoA, 3-hydroxy-3-methylglutaryl–CoA; IV, intravenous; NICU, neonatal intensive care unit; NPO, nil per os.

Kaposiform lymphangiomatosis is a rare lymphatic anomaly characterized by malformations in lymphatic channels affecting multiple organs, most commonly the lungs, mediastinum, and heart.^7^ The cause of kaposiform lymphangiomatosis is unknown, but it does not appear to be inherited.^7^ Symptoms vary by organ involvement and may include pleural and pericardial effusions, bone pain, genital swelling, and consumptive coagulopathy, occurring at a median of 6.5 years.^7,8^ Diagnosis with imaging and biopsy is often delayed because of nonspecific symptoms and limited access to care.^8^ Treatment includes immunosuppression, targeted therapies, and supportive care of coagulopathies and other symptoms, but the prognosis is poor, with an average survival of 2.75–6.7 years from diagnosis because of cardiorespiratory failure or coagulopathy.^7^ There are no known reports of pregnancy in kaposiform lymphangiomatosis.

We present a case of pregnancy in a patient with both HMG-CoA deficiency and kaposiform lymphangiomatosis, resulting in the delivery of a live term infant by vaginal birth.

## CASE

A 24-year-old woman with known HMG-CoA lyase deficiency and kaposiform lymphangiomatosis presented to the emergency department at 6 weeks of gestation with severe vomiting, dehydration, and metabolic acidosis. She was admitted to the antepartum service, where she received aggressive hydration, l-carnitine, and electrolyte repletion. Because of persistent nausea and vomiting, peripheral parenteral nutrition was initiated. After extensive counseling on the risks of poor maternal and fetal outcomes, she chose to continue the pregnancy. A multidisciplinary team, including obstetricians and specialists from maternal–fetal medicine, genetics, and hematology, convened to develop a detailed care plan emphasizing the prevention of hypoglycemia. Sirolimus, her maintenance therapy for kaposiform lymphangiomatosis, was discontinued because of limited safety data during early pregnancy. Her kaposiform lymphangiomatosis remained symptomatically stable during admission. She was discharged on hospital day 5 with a specialized nutritional regimen, including dextrose, protein, and lipids for total parenteral nutrition, and instructed to follow up closely with her care team outpatient.

After improvement in oral intake, total parenteral nutrition was discontinued in the second trimester. Sirolimus was restarted at 1 mg daily at 14 weeks and increased to 1 mg twice daily at 20 weeks because of increasing shortness of breath. Fetal anatomy was unremarkable.

At 28 weeks, she was diagnosed with iron-deficient anemia and received an intravenous iron infusion. Because of her high carbohydrate intake and inability to fast, she did not undergo standard gestational diabetes mellitus testing and was diagnosed with gestational diabetes mellitus on the basis of elevated blood glucose values while her blood sugar was monitored four times daily. Glycemic management was challenging because of inconsistent meals; insulin was avoided as a result of concerns for hypoglycemia. At 32 weeks, fetal growth restriction was diagnosed on the basis of fetal abdominal circumference measuring at the fourth percentile. She also developed thrombocytopenia with the lowest platelet count of 119. Sirolimus was further increased to 1.5 mg twice daily because of worsening cough and shortness of breath. Outpatient fetal surveillance was performed twice weekly. Given concerns about the progression of her kaposiform lymphangiomatosis, fetal growth restriction, and challenges in achieving glycemic control, labor was induced at 37 weeks.

Intrapartum, she received 6 mg/kg/h of carbohydrates through dextrose-containing intravenous fluids. Blood glucose was monitored every 4 hours during latent labor and hourly during active labor. After 20 hours of labor induction, she delivered a male neonate weighing 2,790 g with Apgar scores of 8 at 1 minute and 9 at 5 minutes. Throughout labor, her blood glucose remained between 93 and 132 mg/dL. On postpartum day 1, she received cabergoline for lactation suppression after shared decision making regarding the metabolic stress of lactation. She met postpartum milestones and was discharged home on postpartum day 2.

The patient's infant was readmitted to the neonatal intensive care unit on postpartum day 3 for hyperbilirubinemia and received phototherapy until postpartum day 7. The patient's postpartum course was otherwise complicated by sepsis caused by coronavirus disease 2019 (COVID-19) and superimposed pneumonia requiring a 4-day admission on postpartum day 28. At the time of her 6-week postpartum visit, she was doing well without complaints. Written informed consent was obtained from the patient for publication of this case report.

## DISCUSSION

In the seven reported cases of pregnancy in patients with HMG-CoA lyase deficiency, metabolic decompensation, most commonly in the first trimester and intrapartum, was the most serious complication.^4,5^ Even mild nausea and decreased appetite, commonly experienced in early pregnancy, can trigger metabolic decompensation, characterized by laboratory abnormalities, including hypoglycemia without ketosis, hyperammonemia, and ultimately metabolic acidosis, which can be fatal.^1^ Individuals with HMG-CoA lyase are particularly vulnerable to metabolic decompensation during pregnancy because of increased production of ketone bodies in pregnant patients during fasting, occurring hours earlier than in nonpregnant individuals.^4^ Other obstetric complications noted included cesarean delivery for maternal exhaustion, postpartum hemorrhage, and cardiovascular instability.^5^

To the best of our knowledge, there are no prior peer-reviewed reports of pregnancy in a patient with kaposiform lymphangiomatosis. Contemporary kaposiform lymphangiomatosis literature outlines an aggressive thoracic lymphatic anomaly with hemorrhagic complications and evolving targeted therapies (eg, sirolimus, mitogen-activated protein kinase inhibition), but pregnancy-specific data are absent. Management in our case was therefore extrapolated from general kaposiform lymphangiomatosis care and from limited pregnancy experience with sirolimus in other disorders.^9^

Pregnancy in the setting of dual diagnoses of kaposiform lymphangiomatosis and HMG-CoA lyase deficiency represents an exceptionally high-risk scenario with distinct but overlapping threats to maternal and fetal health. Kaposiform lymphangiomatosis confers risks of progressive respiratory compromise and severe hemorrhage attributable to consumptive coagulopathy, whereas HMG-CoA lyase deficiency predisposes to metabolic decompensation during catabolic stress, fasting, or labor. Fetal risks include fetal growth restriction, preterm delivery, and potential consequences of in utero exposure to sirolimus in kaposiform lymphangiomatosis, as well as the risk of inherited metabolic disease in HMG-CoA lyase deficiency, depending on the father's carrier status (Table 1). Optimal management requires strict avoidance of fasting, vigilant maternal–fetal surveillance, delivery in a tertiary center with access to both metabolic and hematologic expertise, and preparation for massive transfusion and metabolic crisis prevention. Finally, our patient's case highlights the critical importance of multidisciplinary coordination to maximize the chances that these individuals and their neonates can safely navigate the vulnerabilities of pregnancy and achieve positive pregnancy outcomes.
